# Defining the Diffusion in Model Membranes Using Line Fluorescence Recovery after Photobleaching

**DOI:** 10.3390/membranes10120434

**Published:** 2020-12-17

**Authors:** Jakob L. Kure, Camilla B. Andersen, Thomas E. Rasmussen, B. Christoffer Lagerholm, Eva C. Arnspang

**Affiliations:** 1 SDU Biotechnology, Department of Green Technology, University of Southern Denmark, Campusvej 55, 5230 Odense M, Denmark; jlk@igt.sdu.dk (J.L.K.); caba@igt.sdu.dk (C.B.A.); 2 MEMPHYS, Department of Physics, Chemistry and Pharmacy, University of Southern Denmark, Campusvej 55, 5230 Odense M, Denmark; t.e.r@me.com (T.E.R.); christoffer.lagerholm@imm.ox.ac.uk (B.C.L.); 3 Wolfson Imaging Centre Oxford, MRC Weatherall Institute of Molecular Medicine, University of Oxford, Headley Way, Oxford OX3 9DS, UK

**Keywords:** line FRAP, synthetic membrane, diffusion, plasma membrane lipids, microscopy

## Abstract

In this study, we explore the use of line FRAP to detect diffusion in synthetic lipid membranes. The study of the dynamics of these membrane lipids can, however, be challenging. The diffusion in two different synthetic membranes consisting of the lipid mixtures 1:1 DOPC:DPPC and 2:2:1 DOPC:DPPC:Cholesterol was studied with line FRAP. A correlation between diffusion coefficient and temperature was found to be dependent on the morphology of the membrane. We suggest line FRAP as a promising accessible and simple technique to study diffusion in plasma membranes.

## 1. Introduction

**Figure 1:**
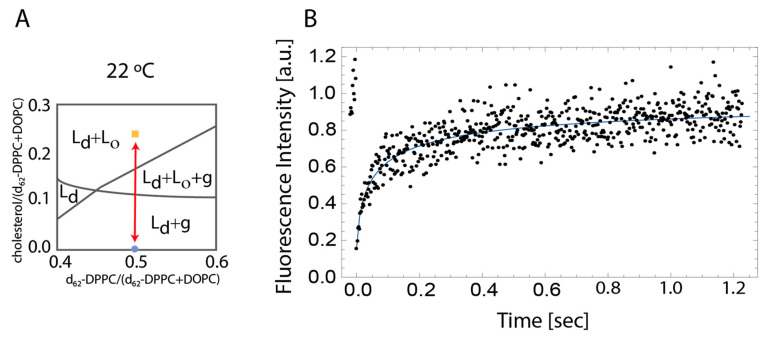
(**A**) Schematic indicating the different equilibrium state at 22 °C between the analyzed membranes adapted from Marsh [9] (**B**) Representative FRAP recovery curve at 22 °C for 2:2:1 DOPC:DPPC:Cholesterol membrane. The blue curve is fitted by using the line FRAP equations (see Equations (1)–(3).

The descriptions of the fluid-mosaic model [1,2] led to an increased focus on understanding the lipid dynamics in biological membranes and gave rise to multiple ways of investigating these; a very popular technique is the use of synthetic membranes. Several synthetic membrane systems have been developed to understand the basic lipid physics in biological membranes. Many of the membrane types have succeeded in showing differences between the different phases in the membrane [3,4,5]. When working with synthetic membranes, the composition and behavior are better controlled, allowing for a more consistent investigation of the feasibility of analytical methods [6,7]. When designing a synthetic membrane system, it is very important to use the relevant equivalence diagrams, with the most effective being the ternary equivalence diagrams. It should be taken into consideration that these diagrams are temperature dependent. Often it is only a small region of the equivalence diagram that results in the desired membrane properties. A common lipid mixture when working with synthetic membranes is a mix of DOPC (1,2-dioleoyl-sn-glycero-3-phosphocholine), DPPC (1,2-dipalmitoyl-sn-glycero-3-phosphocholine) and cholesterol. This mixture is ideal as a synthetic membrane system as it has a relatively large area in the equivalence diagram that fulfills the desired similarities to real plasma membranes [8,9]. A schematic of the relevant area of the equilibrium is shown in Figure 1A.

A direct correlation between the behavior of synthetic and cell membranes should be made with care. The reason being that cell membranes are by nature a lot more complex than synthetic membranes, due to a significantly larger range of lipids and proteins present. This is also the reason for the continued development of more complex synthetic membrane systems [10]. An example of this is that the membrane cholesterol content can vary up to 2.5 fold between cell types, which is difficult to take into account in synthetic membranes [11].

Fluorescence recovery after photobleaching (FRAP) was invented in the 1970s as a tool to study plasma membrane dynamics [12,13]. The evolution of fluorescence imaging in cells and plasma membranes has made the FRAP technique an extensively used tool to study plasma membrane dynamics. FRAP functions by applying a high intensity laser on a spot of a fluorescent labelled sample. Due to the high intensity of the laser, all fluorophores will become irreversibly bleached and the measured fluorescence intensity will be reduced significantly. As time progresses, it can be observed that the fluorescence intensity is recovering in the bleached area. This is because of the particle flow and/or diffusion, where some of the bleached particles will move away from the bleached area, while some of the non-bleached particles will move into the bleached area. After a certain time, depending on the speed of diffusion or flow, the fluorescence intensity will be balanced. There is, however, a fraction of fluorescently labelled immobile particles that become bleached, which results in a weaker fluorescence recovery of the bleached area. The weakened recovery is highly dependent on the fraction of immobile particles.

FRAP has previously shown the ability to define diffusion in both synthetic and cellular membranes [14]. However, the invention of super-resolution techniques like stimulated emission depletion microscopy (STED) [15] and photoactivated localization microscopy (PALM) [16] have gained a lot of popularity within the plasma membrane research field, as more precise methods for determining motion have been developed for these microscopic techniques. This has led to FRAP being pushed a bit into the background because the bleached area is larger. Consequently, this resulted in a slow-down in the development of more advanced approaches to FRAP analytical techniques. FRAP, however, is still a very relevant technique, as not all researchers have access to super-resolution microscopy techniques. Further, there are still a lot of great extensions of FRAP available to define the diffusion coefficient, including empirical, closed form and transform model approaches [17]. The empirical models are usually a fit to the observed fluorescence recovery, e.g., an exponential fit of the fluorescence recovery [17]. The closed form models use theoretical models to quantitively describe the diffusion coefficient. These closed models will end up with a single expression describing only the recovery process of the FRAP experiment; examples of these are the uniform disc model [18], line FRAP [19] and the rectangle FRAP [20]. Lastly, the transform models are based on a transformation of the FRAP data by, e.g., Fourier transformation [21] or Hankel transformation [22]. After the data have been transformed, the diffusion can be determined, without the assumptions that are used in the empirical and closed models. For some of these models, the data acquisition might vary, as the bleaching pattern will be different depending on the derived data analysis. This is the case for the closed form model called line FRAP [19]. As the name reveals, the bleaching happens in a straight line, instead of the circular shape of regular FRAP. Due to this change in shape, Line FRAP allows for faster scanning and makes it possible for more localized identification of diffusion coefficients compared to regular FRAP [19]. The model is based on the following equation, which is derived in Braeckmans et al. [19].

(1) $$\frac{F\left(y,t\right)}{F_{0}}=\overset{+\infty }{\underset{n=0}{\sum }}\frac{{\left(-K_{0}\right)}^{n}}{n!}\cdot r_{0e}\cdot {\left(n\cdot r_{0c}^{2}+\left(a_{n}-n\right)\cdot r_{0e}^{2}\right)}^{-\frac{1}{2}}$$

where *F* corresponds to the fluorescent intensity and *F*_0_ is the fluorescent intensity before bleaching. *K*_0_ is the bleaching parameter, *n* is the number of data points, *r*_0*e*_ and *r*_0*c*_ are the bleaching and imaging resolution, respectively. The parameter *a_n_* is calculated as seen below, where *D* is the diffusion coefficient and *t* is the time since photobleaching.

(2) $$a_{n}=1+n\cdot \left(1+2t\cdot \frac{4D}{r_{0e}^{2}}\right)$$

The line FRAP equation can be substituted into the following equation to get a multi-component expression, which is used to extract the diffusion coefficient from the fit.

(3) F(y,t)=F(y,0)+k·(F(y,t)−F(y,0))

The aim of this study is a proof of principle that line FRAP is an applicable technique for identification of the diffusion coefficient in synthetic lipid membranes. This is demonstrated using two synthetic membranes prepared from DOPC, DPPC and fluorescent labeled phosphocholine with and without cholesterol. The study of the two membranes shows that line FRAP can detect differences in diffusion in the membrane that are dependent on the temperature and the membrane composition.

## 2. Materials and Methods

Stock solutions of DOPC, DPPC and cholesterol (Avanti Lipids, Alabaster, AL, USA) were prepared in concentrations of 10 mM in a 9:1 chloroform and acetonitrile solution. The solutions were then mixed into two different solutions, 1:1 DOPC:DPPC and 2:2:1 DOPC:DPPC:Cholesterol, and the fluorescent dye NBD-PC (Invitrogen, Waltham, MA, USA) was added to a concentration of 0.5 molar percent of the total lipids. Coverslips were washed by mounting them on Teflon holders and put into a 12% hydrogen peroxide + 12% ammonia in a milli-Q water solution (Sigma-Aldrich, St. Louis, MO, USA). The solution was heated and boiled until bubbles stopped appearing. Then, the holder was removed from the solution, washed with milli-Q water 5 times, and left to dry at 90 °C for two hours.

To coat the coverslips with a lipid membrane, the dried coverslip was placed under vacuum in a spin coater (KW-4A Spin Coater, Chemat Scientific, Northridge, CA, USA). Then, 50 µL of the lipid solution was added in the middle of the coverslip and the spin coater was turned on for 3 s at 500 rpm followed by 40 s at 3000 rpm. Afterwards, the coverslips were put into a desiccator for 24 h to ensure complete removal of the solvent.

The coverslip with an applied spin coated membrane was then mounted on a stage heater (Warner TC-344D, Warner Instruments, Holliston, MA, USA) fitting the used confocal microscope (Zeiss LSM 5, Carl Zeiss, Oberkochen, DE, Germany). PBS (Sigma-Aldrich) was then added onto the membrane and it was heated to 60° C, and the membrane was observed. If there was no clear patch of membrane, the PBS was pipetted gently up and down to remove the top bilayer, then the PBS was removed, and some new PBS was added. This pipetting process was repeated until a clear patch of membrane was observed. When the membrane was at a satisfactory state, it was imaged using the line FRAP technique with an ROI of 100 × 1 pixels. The 1:1 DOPC:DPPC membrane was imaged outside domain like areas, which was not done in the 2:2:1 DOPC:DPPC:cholesterol due to the nature of this membrane.

After imaging, the data were fitted with a double exponential function, which was used to extract two diffusion coefficients. All data analysis was performed in Mathematica (Wolfram Mathematica, Wolfram Research, Champaign, IL, USA).

## 3. Results and Discussion

Line FRAP images were acquired for the two different synthetic membrane systems. Assuming irreversible bleaching, the images were analyzed using the multi-component function (see Equation (3)), resulting in two distinct diffusion coefficients. An exemplified fit of the double exponential function is found in Figure 1B.

**Figure 2:**
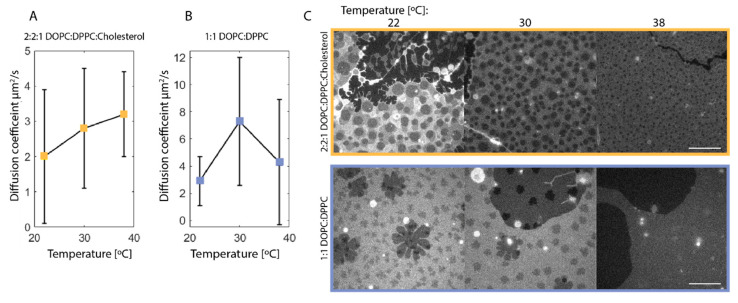
(**A**,**B**): Diffusion coefficient of NBD-PC in synthetic membrane extracted from line FRAP with the multi-component function in Equation (3) at 22 °C (**A**: *n* = 12, **B**: *n* = 12), 30 °C (**A**: *n* = 10, **B**: *n* = 3), and 38 °C (**A**: *n* = 4, **B**: *n* = 12), where n is the number of line scans. Note that the y-axis varies between (**A**,**B**). Error bars indicate standard deviation. There is an increase in diffusion coefficient with an increasing temperature for the 2:2:1 DOPC:DPPC:Cholesterol membrane. For the 1:1 DOPC:DPPC synthetic membrane, there is no clear tendency in terms of increasing temperature, which is likely due to the heterogeneity of the membrane type, as seen in (**C**). (**C**): Confocal images of the synthetic membranes at 22, 30 and 38 °C. Top: 2:2:1 DOPC:DPPC:Cholesterol synthetic membrane showing a homogeneous distribution of domains and a reduction in the total domain area with increasing temperature. Bottom: 1:1 DOPC:DPPC synthetic membrane shows a similar reduction in domain area; however, the membrane is heterogeneous with gel formation. It appears, however, that the gel phase has disappeared at 38 °C. The scalebar is 10 µm.

The first diffusion coefficient measured from all line FRAP data was 5 to 10-fold higher than in comparable synthetic membranes, reaching from 16 to 45 µm^2^/s [23] (data not shown); this is expected to be due to free fluorophores in the buffer solution surrounding the membrane and will therefore be neglected in the further data analysis. The second diffusion coefficient shows a correlation to the temperature in the synthetic membrane containing 2:2:1 DOPC:DPPC:Cholesterol. The increase in diffusion coefficient with temperature is expected as a reduction in lipid domains is observed with increasing temperatures, as illustrated in Figure 2C. It is further observed that this synthetic membrane system is very uniform compared to the 1:1 DOPC:DPPC membrane system where smaller similar domains are observed, but this system shows larger flowery looking domains as well (Figure 2C). The flowery domains arise from gel formation that is expected from this system, as it is in a different equilibrium state than the 2:2:1 DOPC:DPPC:Cholesterol according to the equivalence diagram in Figure 1A. The formation of the flowery domains corresponds well with different studies using the same membrane composition [24,25]. The flowery domains in the 1:1 DOPC:DPPC membrane interfere with the diffusion behavior in the membrane, which explains the fact that the diffusion coefficient does not show any correlation to the temperature.

The diffusion coefficient behaves as would be expected for the analyzed synthetic membrane systems. Prior to these experiments, a variant of line FRAP was successfully used on cyanobacteria [26,27]. This knowledge, combined with the data from this work, indicates that the line FRAP technique has great promise for determining the diffusion coefficient in synthetic membrane systems and thereby indicating the possibility to implement the line FRAP technique into the membrane research of live mammalian cells. When performing line FRAP on live mammalian cells, a high number of cells must be analyzed, as cell to cell variability is to be expected. Line FRAP only requires a laser scanning confocal setup, which makes the technique widely accessible since most imaging facilities have this setup. This gives Line FRAP an advantage over more advanced techniques such as fluorescence correlation spectroscopy (FCS) and stimulated emission depletion microscopy-FCS, and also provides the opportunity to measure diffusion coefficient in the plasma membrane [28]. Based on the results from this paper, it should be possible to use the line FRAP to study the diffusion of different particles in live mammalian cells, resulting in a more precise analysis due the nature of line FRAP. A more precise analysis might reveal undiscovered intercellular processes and provide new insights into cell morphology.

## 4. Conclusions

The study shows a correlation between the diffusion coefficient measured with line FRAP of the synthetic model membranes and the observed morphology of the membrane. In a mixture of 2:2:1 DOPC:DPPC:Cholesterol, domains appeared uniformly across the synthetic membrane. The line FRAP analysis showed an increase in diffusion coefficient with temperature, as is expected as the domain fraction decreases with increasing temperature, making more room for free diffusion. The same trend was not observed in the 1:1 DOPC:DPPC mixture, where there was no correlation between the temperature and the diffusion coefficient. The lack of correlation is due to the fact that the mixture was in a different equilibrium state, giving rise to gel formation, and therefore did not behave in the same manner. This observation is supported by the lack of uniformity observed for these synthetic membranes. Based on these experiments with synthetic membranes, line FRAP shows great promise as a technique to identify the diffusion coefficient in biological membranes. The possibility of using line FRAP in live mammalian cells should be further investigated.
